# Internal Damage Detection of Composite Structures Using Passive RFID Tag Antenna Deformation Method: Basic Research

**DOI:** 10.3390/s21248236

**Published:** 2021-12-09

**Authors:** Pavol Pecho, Michal Hrúz, Andrej Novák, Libor Trško

**Affiliations:** 1 Air Transport Department, University of Zilina, Univerzitna 8215/1, 010 26 Zilina, Slovakia; michal.hruz@stud.uniza.sk (M.H.); andrej.novak@fpedas.uniza.sk (A.N.); 2 Research Centre, University of Zilina, Univerzitna 8215/1, 010 26 Zilina, Slovakia; libor.trsko@uniza.sk

**Keywords:** RFID, tensile test, fibreglass, aircraft maintenance, failure detection, crack detection, composite structure

## Abstract

This manuscript deals with the detection of internal cracks and defects in aeronautical fibreglass structures. In technical practice, it is problematic to accurately determine the service life or MTBF (Mean Time Between Failure) of composite materials by the methods used in metallic materials. The problem is mainly the inhomogeneous and anisotropic structure of composites, possibly due to the differences in the macrostructure during production, production processes, etc. Diagnostic methods for detecting internal cracks and damage are slightly different, and in practice, it is more difficult to detect defects using non-destructive testing (NDT). The article deals with the use of Radio frequency identification (RFID) technology integrated in the fibreglass laminates of aircraft structures to detect internal defects based on deformation behaviour of passive RFID tag antenna. The experiments proved the potential of using RFID technology in fibreglass composite laminates when using tensile tests applied on specimens with different structural properties. Therefore, the implementation of passive RFID tags into fibreglass composite structures presents the possibilities of detecting internal cracks and structural health monitoring. The result and conclusion of the basic research is determination of the application conditions for our proposed technology in practice. Moreover, the basic research provides recommendations for the applied research in terms of the use in real composite airframe structures.

## 1. Introduction

To begin with, the superior mechanical properties of composite materials guarantee their application in numerous industry sectors. Unlike the lightweight metal alloys, the advantage of composites lies in their weight saving potential, high strength, and stiffness with the ability to be specifically manufactured or tailored with the anisotropic properties for their specific applications [1,2]. In general, the composite structure is defined by the combination of two different materials representing the matrix and reinforcing element, together enhancing the overall structural performance [3]. The most applied composites for primary and secondary structures of commercial and military aircraft in the aviation are carbon fibre reinforced polymers (CFRP) and glass fibre reinforced polymers (GFRP). These composites contain extremely thin carbon or glass fibres in polymeric matrices [2]. Today’s commercial aircraft structures, mainly due to the reliability and cost-effectiveness consists mostly of composites usually applied on exposed, load carrying surfaces which are prone to damage thus requiring a regular maintenance [4]. Airbus and Boeing, the world’s leading aircraft manufacturers, estimated a doubling of the aircraft fleet until the year 2035 compared to 2016 [5]. Modern widebody aircraft, such as the Boeing 787 Dreamliner and the Airbus A350, are made of more than 50% carbon fibre composite [6]. Moreover, each generation of new aircraft built by Boeing had an increased percentage of the composite material usage [7,8]. Airbus also found the greater use of composites on their planes beneficial. For instance, the increased usage of composite materials on the Airbus 350 resulted in a 50% reduction of required structure maintenance tasks, and additionally led to the extension of the schedule for airframe checks for the jetliner to every twelve years, in contrast to A380, for which these checks are scheduled every eight years [9]. Both mentioned manufacturers show a continuing trend toward greater use of composite materials on their aircraft thanks to their long-term benefits.

Regarding the overall effectiveness, today’s trend in aviation focuses on replacing regularly performed preventive maintenance strategies with an advanced predictive (PdM) and proactive maintenance (PaM) procedures as these save time, workload, and most importantly costs, while maintaining or even improving the current safety levels of maintenance and repair organisations (MRO’s). This trend impacted many sectors, especially the aircraft maintenance sector, where the emphasis is placed primary on reliability while maintaining the cost efficiency demands its inclusion promptly. The predictive maintenance, as it names suggests, aims primary at forecasting or predicting of pending system failure or failure of one of its parts based on real-time monitoring of significant operational parameters (such as temperature, vibration, pressure, or applied load) defining their actual technical condition. Together with proactive maintenance, which uses analytics to spot and track trends, the two strategies can accurately predict when failure may occur and according to that plan the maintenance when it is needed. This reduces the costly unexpected downtimes as parts are replaced according to their actual operational condition and allows maximising their service life, unlike in case of preventive strategies, as these parts are replaced either too early or too late [10,11].

Obviously, the growing use of composites in the field of aerospace industry represents new challenges for MROs in the field of reliable and rapid damage detection, and subsequent evaluation of their severity, as these materials differ significantly from homogeneous metals or alloys. The structural damage can occur during their production in the form of material contamination, porosity, delamination of matrix layers, or during the operation of the aircraft itself. Despite their strength, the composite materials are susceptible to structural damage when subjected to excessive stress or low-velocity impact (LVI), which are common phenomena occurred during aircraft operation, resulting in matrix cracks or fibre matrix debonding. Regarding the correct stress distribution, thus achieving the best possible properties, the correct ply orientation of composite laminates is crucial. In addition to mechanical damage, exposed composite parts of the aircraft structure are also affected by weather conditions in the form of the lightning strikes or the large hail impact [7]. Although such damage may not be directly visible, it may cause damage to the coating, or even irreversible structural damage, which must be considered for safety reasons. Ultimately, these anomalies will drastically affect the mechanical properties of the composite materials [12] and the overall reliability, safety, and airworthiness of the aircraft.

### 1.1. The Role and Types of Non-Destructive Testing

The non-destructive testing (NDT) plays a significant role in the field of the aircraft maintenance, as it is used to inspect aircraft and their parts in a safe, reliable, and cost-effective manner without damaging or affecting their future usefulness. The NDT methods have been active areas of research for many years. Generally, the NDT is usually applicable in production phases to ensure there are no hidden defects and damage presented in material structures, but it is also used during the aircraft operation phase as well representing a maintenance tool for detecting abnormalities, such as cracks, corrosion, or other forms of degradation that can cause system or its related parts to fail [13,14]. The aerospace industry has always been the leader in the development of structural health monitoring (SHM) systems, which are key technologies to ensuring the structural integrity of future aircraft structures. There are many types of NDT methods used in the aircraft maintenance sector, but not all are suited for SHM applications due to integration and cost difficulties [15]. In addition, traditional metals-based NDT methods without additional modifications are inappropriate and often misleading when applied to anisotropic and inhomogeneous composite materials [2]. In contrast to composites, the damage and failure in case of metallic structures is well-researched and understood. The most frequent damage is in the form of fatigue cracks, which are additionally spreading under cyclic loading. In case of composites, the damage can occur in many more ways, as they behave differently under tension as they do in compression [16]. Among the NDT methods for composites, pulsed eddy current (PEC) and eddy current pulsed thermography (ECPT) have proved successful for the detection of internal damage, such as cracks caused by fatigue and corrosion, while providing good resolution, sensitivity, and reliability [17,18]. However, these methods have their limitations, which do not allow their effective application on larger structures, since these methods involve high labour and wiring costs [18,19]. Furthermore, relevant outputs in a good resolution are range and power limited.

### 1.2. Damage Detection and Control Approaches for Larger Structures

To effectively detect and at the same time reduce the time required for damage detection and evaluation, several SHM methods have been developed using sensor equipment in earlier stages (using guided wave ultrasonics) and progressive RFID technology over time. The guided wave technique does not provide accurate remaining thickness information and it is best complemented by point measurements at selected locations. Another issue is that the SHM transducers must survive in operational conditions, which is particularly difficult at high temperatures [20]. Ihn and Chang, in 2004 [21], announced a diagnostic technique for monitoring crack growth in metallic structures using built-in piezoelectric sensor/actuators. The technique monitored the expansion or growth of the crack and its subsequent evaluation. The results of this technique showed a good correlation with actual fatigue crack growth obtained from visual inspection. To improve efficiency and gain a competitive advantage, major airports and airlines adopted RFID technology, which was developed and used as an early-stage technology on the Internet of Things (IoT) [22]—a core technology of the fourth industrial revolution. The airports and airlines used RFID in various processes [23]: baggage handling and tracking [24], monitoring of individual aircraft parts and supply chains [25,26], and as health monitoring systems [26,27,28,29].

Previous methods in the case of monitoring large structures have involved the use of numerous sensors, forming large-scale sensor networks requiring long cabling. During the time, these were replaced by wireless battery-powered sensors, but these were twice as expensive and non-ecological option as cable equivalents. In the case of applications on larger structures, such as aircraft structures, the challenge lied in design a network of small wireless, sufficiently accurate, reliable, and low-cost sensors. At the same time, these sensors should be able to be placed in hard-to-reach places, with the possibility of passive autonomous sensing, and able to communicate. This is the main reason why applications of RFID in the aviation sector are still growing.

### 1.3. Radio Frequency Identification Technology (RFID)

**Figure 1:**
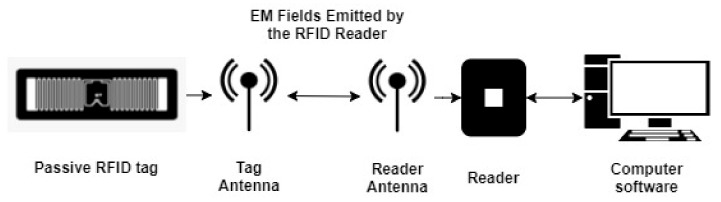
Scheme of information transmission from passive RFID Tag to RFID Reader.

The RFID system consists of RFID tags, RFID reader, and software for managing the obtained information. RFID uses electromagnetic fields to automatically identify and track tags attached to objects. When triggered by an electromagnetic interrogation pulse from a nearby RFID reader device, the information from the RFID tag is acquired and transmitted to the software, as is shown in Figure 1. The RFID tags are divided into passive (without the battery, powered by electromagnetic waves provided by a reader) or active type (typically powered by a battery) [30]. The crucial properties of RFID tags are the facts that they do not need line-of-sight alignment and the RFID reader can read multiple tags simultaneously. The passive RFID tags with their low-energy consumption, thus low environmental impact, with constantly increasing interrogation distance and wireless characteristics, represent an ideal option for structural health monitoring applications.

The use of RFID technology in aviation can be divided into two basic groups:

1. RFID devices that affect or may affect the safety and airworthiness of the aircraft.
2. RFID devices that are related to the operation of the airline and aircraft maintenance but do not affect the safety and airworthiness of the aircraft.

**Figure 2:**
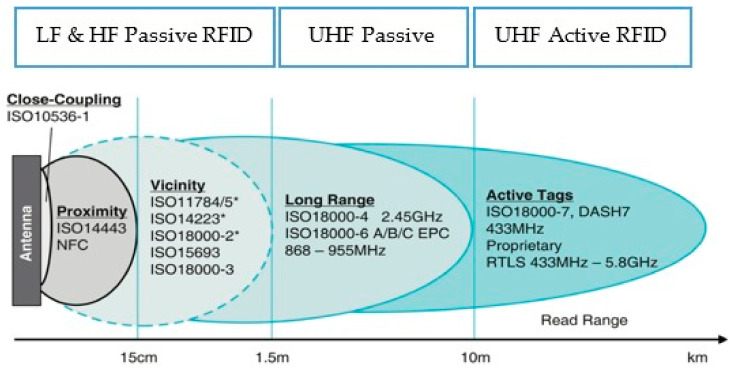
Distribution of RFID technologies in terms of reading device range and frequency (* 125–134 kHz); Source: [31].

Passive Low Frequency (LF) and High Frequency (HF) RFID use the magnetic coupling of the electromagnetic field to transmit power and data. Ultra-High Frequency (UHF) passive and active RFID are based on the e-field coupling. The type of connection is affected by factors such as reading distance, data rate, and environmental resilience. Figure 2 shows how the four standards (LF, HF, UHF, Active UHF RFID) relate to each other, in terms of range and frequency used to read and power the device. Near Field Communications (NFC) is a subset of RFID technologies and is important for secure communication between devices over short distances [31].

### 1.4. Damage Types of Composites

It is estimated that the fatigue, corrosion, and associated cracks are one of the most common types of damage on aircraft structures, as about 60% of the total failures on aircraft is caused by fatigue and 16% by corrosion [32,33]. Daily inspection of damages, such as corrosion and cracks of composite structures is a time-consuming process [12] where the technician must measure the extent of corrosion and the severity of the cracks, and this time increases because the technician’s activities must be additionally verified by a certified mechanic [27].

In laminated composites, there are three main damage or failure modes distinguished:

- Intralaminar (intra-ply or intralaminar cracks);
- Interlaminar (delamination);
- Translaminar (fibre failure or breakage) [34,35].

These types of damage depend on the type and direction of reinforcement, and in addition to that, also on the direction and type of mechanical stress. In general, the composite damage has a constant course depending on the extent of the mechanical stress. At first, the damage occurs in zones with lower strength, such as the interface between the fibres and the matrix, while this type of damage is called intralaminar cracks. These cracks occur when the stress in the matrix reaches its breaking strain, mainly in areas where the fibres are not oriented in the load axis; thus, they are usually parallel and separated from each other and ultimately have little effect on the final strength of the material [34,35,36,37].

The delamination of composite structures is caused by high intralaminar stresses in conjunction with typically very low through-thickness strength of the laminate, occurring at the interface, between adjacent layers, due to propagation of intralaminar cracks or edge effects caused by the stress. In general, this phenomenon occurs to prevent the distribution of stresses between the layers. Since the fibres lying in the plane of the laminate, they do not provide reinforcement across the thickness, the load in this direction is carried by a relatively weak and often brittle resin matrix. Moreover, it occurs even when the individual layers of the composite have different directions, and thus different stiffness. The delamination of the composite structure causes the stiffness loss, local stress concentration in loading layers, and the local instability, which causes further growth and leads to compressive failure. Ultimately, the delamination leads to the redistribution of structural load paths, leading to structural failure. This damage can be the result of a manufacturing error or impact, and indirectly affects the final structural failure, and thus its service life. With a significant increase of the mechanical stress, translaminar failures, such as fibre breakage and matrix damage occur [34,35,36,37].

Therefore, the above-mentioned statements represent an ample motivation to search for new SHM method for monitoring safety-critical structures over their service life to improve their reliability and availability, as well as reduce their maintenance costs, especially in the field of aircraft maintenance, with its zero tolerance for catastrophic failure. In summary, detection of internal stress and the associated detection of cracks in composite materials in structural levels could primarily increase the overall safety and sustainability of the aircraft airworthiness, while secondarily ensuring a reduction in repair and maintenance costs thanks to its effective predictive and proactive nature. Hence, the presented article aims on design of new SHM methodology for internal stress and damage detection of glass-reinforced composite materials using RFID technology thoroughly described in the following chapters. It is the early detection of material inhomogeneity that will enable the early detection of a potential critical error, while the rapid reading of RFID technology will make the overall maintenance process more efficient. The methodology in the manuscript was the creation of samples that analogously simulated composite structural units of fibreglass aircraft with integrated passive RFID tags. The experimental part of the research was focused on tensile tests with complete destruction of samples, with the behaviour monitoring of materials and RFID tags during the entire time horizon, meaning from zero load to rupture of the sample. The primary result of the experiments was the determination of the appropriate RFID tag type from three different variants that were used.

## 2. Materials and Methods

### 2.1. The Structure of Tested Specimens

In order to create two different specimen types with different structural characteristics for tensile testing, two glass fibre variants of different weighing were used. These two variants of reinforcing elements together with epoxy resin created the composite materials representing our tested specimens: the first type was Aeroglass fabric, weighing 130 g per square meter, with as additional labelling “high strength”. We used the canvas design of the fabric. The second type was a lighter fabric, weighing 80 g per square meter. The difference compared to the first type was a twill design with the same trade name Aeroglass. Both fabrics are intended for common use, and especially in the aerospace industry. A resin and a hardener were used to create a matrix, to ensure the greatest possible degree of similarity with the fibreglass structures of the aircraft. It was an L 285 resin (MGS) type with a 285 MGS hardener mixed in a weight ratio of 100:40 [38].

**Figure 3:**
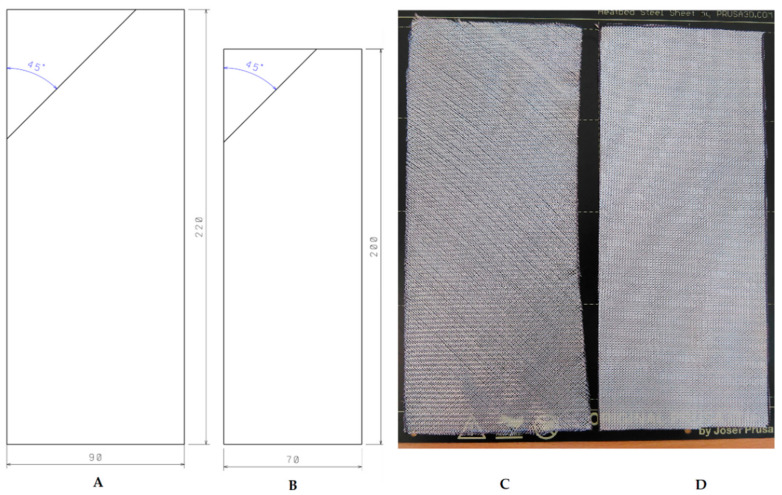
Diagram of sample’s dimensions during manufacture process (**A**), and a final sample size after processing (**B**); Comparison of fibreglass fabric type “80” (**C**) and type “130” (**D**).

During the forming process of individual samples for two different specimens, individual fabric portions (layers) were cut at a 45-degree angle corresponding to a 45-degree ply orientation of fibres, with an overall dimension larger than the final dimension of the samples, as can be seen in Figure 3.

**Figure 4:**
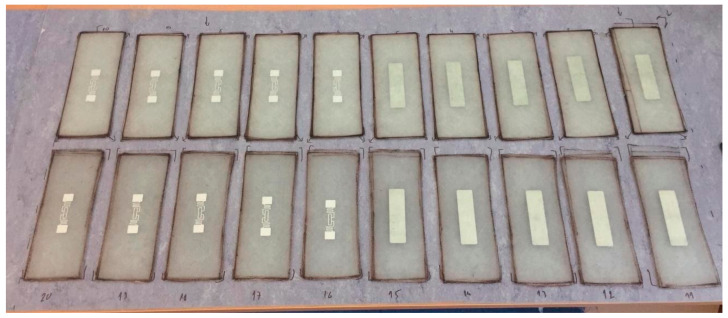
Storage of individual samples during the lamination process on a glass surface.

The default dimensions were 220 × 90 mm and after the samples had hardened (see Figure 3), they were subsequently ground to a final shape of 200 × 70 mm (see Figure 4), and code numbered according to their characteristics which are further defined in following Section 2.2. A total of 10 layers were laminated, with the RFID tag placed between the fifth and sixth fabric layers.

**Figure 5:**
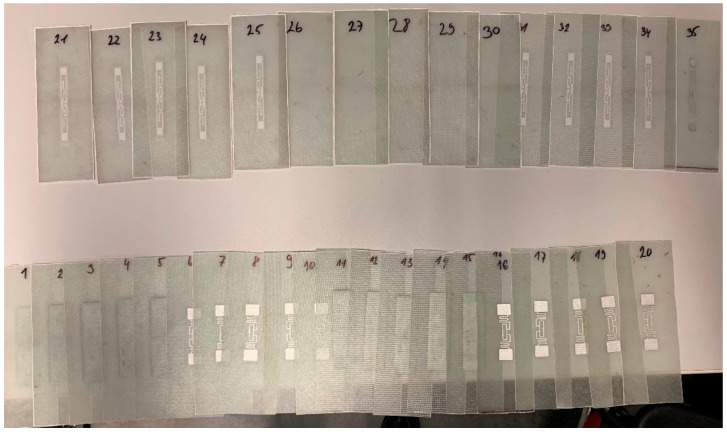
Laminated samples consisting of three series with different passive RFID tags integrated and one series without RFID tags.

The lamination process was conducted on the glass surface without additional vacuuming or pulling on the top. The samples were freely air-dried for 24 h and, after curing, were peeled from the glass surface, and subjected to mechanical treatment to form a unified shape, see Figure 5.

### 2.2. Code Disignation of Tested Samples

In total, 40 samples were examined in the presented experiment, according to the standards of ISO 527-5:2009 (Plastics—Determination of tensile properties—Part 5: Test conditions for unidirectional fibre-reinforced plastic composites). As these were non-standard samples, the use of information from ISO 527-5:2009 had only the character of meeting the shape and aspect ratios of the tested samples. The samples had their own code designation, which corresponds to their three characteristic parameters separated by dashes. The first digit represented the serial number of the sample in the range of 1–40. The second digit represented the type of fibreglass fabric used in two versions for 80 g and 130 g. The last digit represented the type of RFID tag (1, 2, and 3), there was also a 0 digit, which represented samples without RFID tags. Thus, the code number “10-130-1” represents the sample number 10 using a fabric weighing 130 g per square meter and containing the number 1 type of passive RFID tag.

### 2.3. Tensile Tests

**Figure 6:**
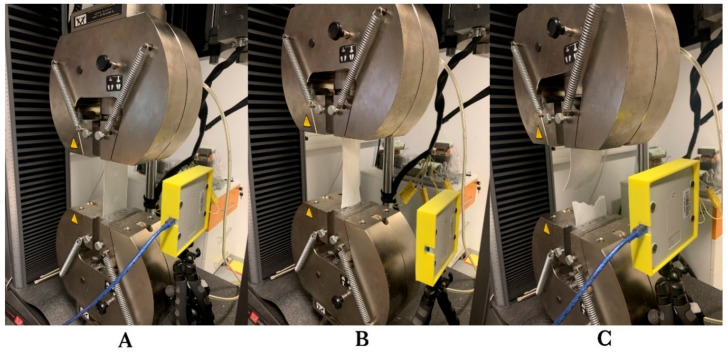
Process of tensile tests of individual laminated samples with passive RFID tags monitored by RFID antenna (Yellow Box), default placement position of the sample clamped in pneumatic flat grips (**A**), course of sample deformation (**B**), and final state after the sample destruction (**C**).

The used device for tensile tests was Instron 5985, with a maximal loading capacity of 200 kN. Tests were carried out at room temperature, with a displacement rate of 1 mm·min^−1^. The displacement rate was chosen to provide reasonable testing time for proper RFID signal reading. Samples were clamped with a clamping pressure of 0.3 MPa (see Figure 6), which did not cause damage to the composite, but still provided sufficient gripping efficiency to prevent the samples from slipping, which might have resulted in showing false elongation values. Prior to gripping, sample protection was always activated to avoid their initial damage due to axial forces caused by the gripping mechanism.

### 2.4. Types of Passive RFID Tags and Antennas

**Figure 7:**
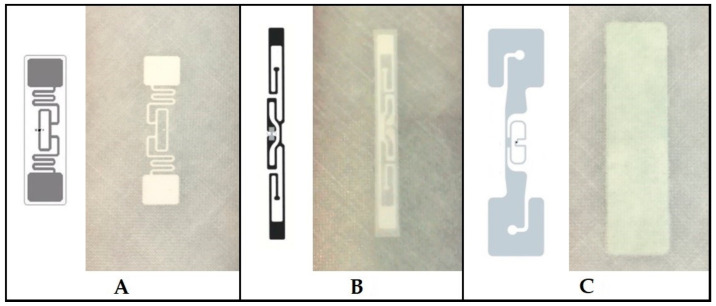
Individual tested RFID tags models: (**A**) AZ9662 H3, (**B**) AD-226iM, (**C**) DogBone and their implementation in laminate structures; Based on [39,40,41].

For the experimental testing of RFID use in air transport, especially in the aircraft maintenance sector, three basic and commonly used types of passive RFID tags were selected. This solution was based on the basic requirement of using the commercially available RFID tags. The selection and specification of these tags were in accordance with the methodology set out in the paper “Implementation of smart technologies into the civil aviation aircraft maintenance process” [31]. The requirements for these RFID tags are operating frequency in the UHF band, long range, Low-mem tags, GS1/EPC Class 1 Gen 2 in accordance with ISO 18000-6C. In this research, following commercially the most often implemented UHF RFID tags in the fields of the logistic and aviation industry were used:

1. RFID tag n. 1 is AZ9662 H3, global operating frequency (860–960 MHz), low mem tags EPC 96-bit, USER 512 bit; antenna dimensions 95 × 8.15 mm; see Figure 7A.
2. RFID tag n. 2 is AD-226iM, global operating frequency (860–960 MHz), low-mem tags EPC 256 bit; USER 512 bit; tag ID (TID) 96 bit; antenna dimensions 93 × 23 mm; see Figure 7B.
3. RFID tag n. 3 is DogBone, global operating frequency (860–960 MHz), no-mem tags EPC 96 bit; antenna dimensions 73.5 × 21.5 mm; see Figure 7C.

For this experiment, RFID tags had to be in accordance with basic requirements for the storage and identification of aircraft parts. The first phase of the experiment was focused only on the basic ability to identify and read the unique number—the tag ID (TID) of the RFID tag—as well as on the ability to communicate in an environment affected by signal reflection and interference. The size and the position of the antenna are one of the essential parameters for the experiment, and each of the selected tags had to have two antennas for short-distance as well as long-distance reception (see Figure 7).

**Figure 8:**
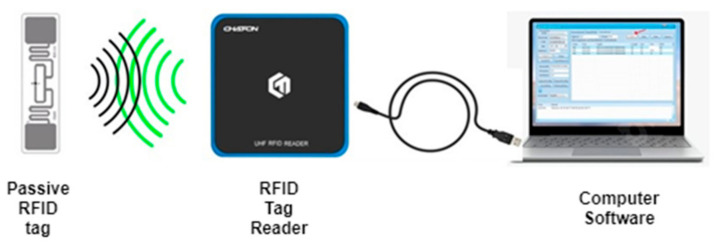
Schematic block diagram of the RFID tag measurement.

The CF-RU5000-USB set-top box (see Figure 8 (RFID Tag Reader)) from the manufacturer CHAFON was using as a scanning and measuring device for reading UHF RFID tags. The transmitting power of the device was set to 17d bm and using the frequencies: 902–928 MHz (US standard) and 860–868 MHz (EU standard). This measurement was assigned a frequency band from 860 to 868 MHz. The device is compatible with ISO 18000-6C (EPC C1G2) with an active antenna size of 100 × 100 mm. The schematic block diagram of the measurement connection is shown in Figure 8. The scanning distance was set to 10 cm from the sample.

**Figure 9:**
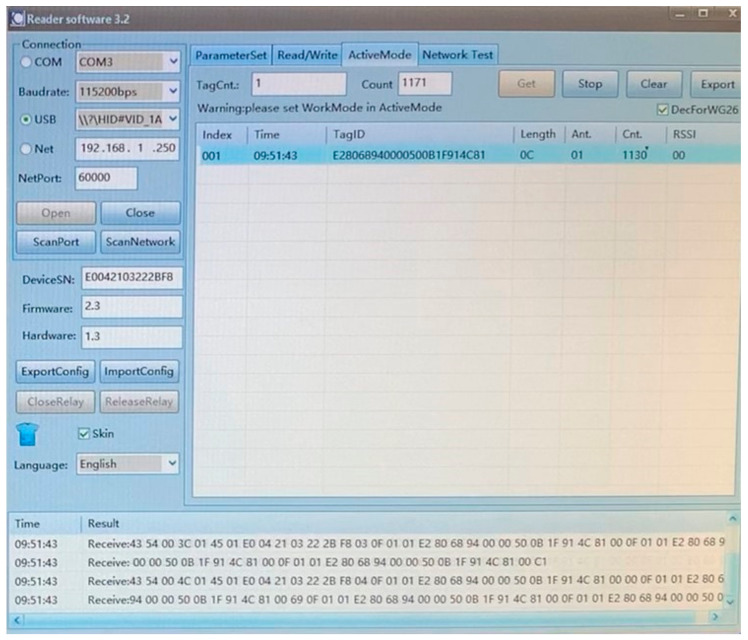
Image taken from recorded footage of communication monitoring between the tag antenna and reader in the Reader Software environment.

**Figure 10:**
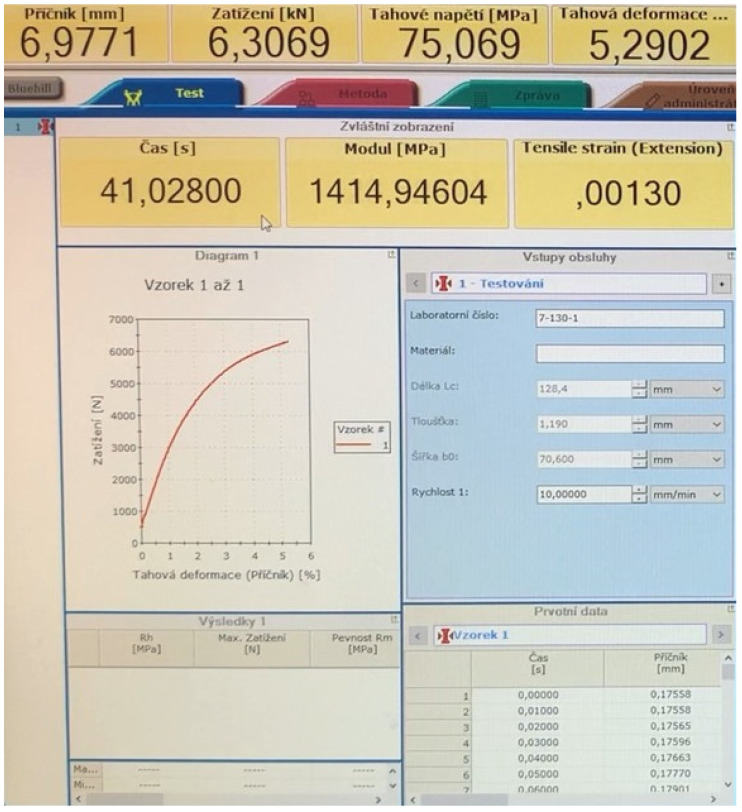
Image taken from recorded footage of tensile testing of sample “7-130-1” in the Bluehill Instron Materials testing software environment.

The scanning system has been set to scan Electronic Identification (EID). Subsequent scanning was done in ActiveMode mode (see Figure 9), which reads the RFID tag at a rate of 10 times per second. The recording of this reading was transferred to a file via Reader Software and subsequently processed together with the tensile test (see Figure 10) of the measured sample (described in Section 2.3). The experiment aimed at the loss of the connection between the RFID Reader and the RFID tag antenna integrated in the tested sample during the tensile test. Secondly, the important index was the transmission termination of RFID based on the deformation and damage to the RFID tag antenna.

## 3. Results

The following section presents the values of individual measurements, divided into four main categories. The individual groups are characterised by the type of RFID tag used, and as was mentioned earlier, the category marked “0” represents a group of samples without an RFID tag integrated in structures. During the tensile tests, the following parameters were evaluated:

- Maximum Load [N];
- Ultimate tensile strength Rm [MPa];
- Proof stress Rp 0.2 [MPa];
- Ductility [%].

From the individual measurements, the parameters for each group were evaluated by an average value, further processed into a form of graphical comparison.

### 3.1. Measurements Using Samples without an RFID Tag

The first analysed group of samples subjected to tensile tests did not have implemented RFID tags in their structures. The group represents a nominal sample for the study of the tags’ effects on the composite samples’ internal integrity. According to Table 1, these were samples number “26-30”, using fabric type “130”, and samples “36-40”, using fabric type “80”. In the case of test sample with a code number “30-130-0”, the measurement was not relevantly recorded, since during the tensile test, an unexpected error occurred, making it impossible to provide relevant data for this measurement. This test represents the only deviation during this tested group category of the experiment.

**Figure 11:**
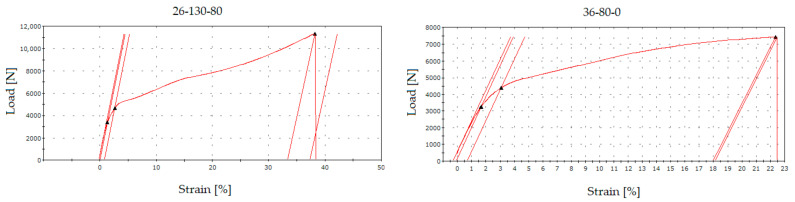
Comparison of tensile diagrams of group “0” for fabrics type “130” and “80”.

Based on the average values of the individual groups, it is obvious that the use of the “130” fabric type shows significantly higher values of the applied load, and in the case of ductility, there was an almost 100% increase compared to the “80” fabric type. In addition to the numerical course, a graph representing the course of the applied load in [N] against strain [%] was generated from each static tensile test. Figure 11 represents the measurement process of Sample “26-130-0” (Figure 11 left) compared to Sample “36-80-0” (Figure 11 right) to capture the differences of the different fabric types.

### 3.2. Measurements Using Samples with RFID Tags: Type No. 1

Compared to the first group of measurements, the following three groups had RFID tags integrated in their structures. The first group is type no. 1. As in the case of group 0, one measurement error occurred in this group, only for sample “9-130-1”. Other samples during the tensile test were scanned by the RFID reader to capture the deformation (moment of damage) of the tag antenna during the test. The total maximum values for the individual samples as well as the average values of the tested groups are shown in Table 2.

**Figure 12:**
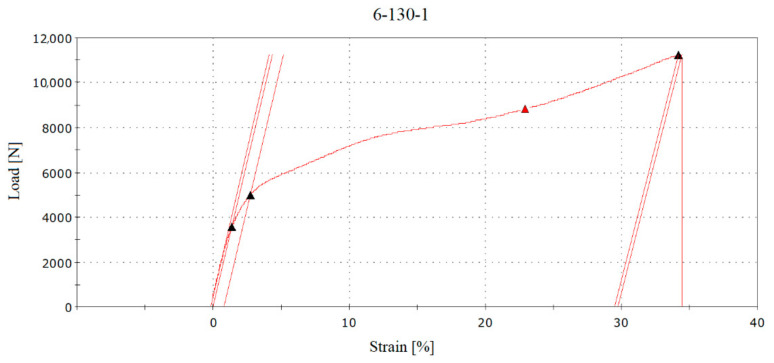
The point of interruption (red triangle) of the RFID tag transmission at approximately a load value of 8700 [N] and a strain of 23.5% with sample no. “6-130-1”.

In the RFID tag type no. 1 group, transmission interruptions were recorded and captured in the graphical representation of the tensile test. Figure 12 shows sample “6-130-1”, where the black triangles represent the boundary areas of the test and the red triangle on the curve represents the point of an interruption of the RFID tag antenna transmission. The interruption values were read from the numerical course of the test, the recording frequency of which was one hundredth of a second. In the case of sample “6-130-1”, the transmission of the RFID tag was interrupted within 178 s from the start of the test. This point corresponded to the values load of 8848 N and strain of 29.8 mm. The percentage evaluation of strains was calculated in graphical form based on the sample dimensions and the distances of the sample grips before the test.

**Figure 13:**
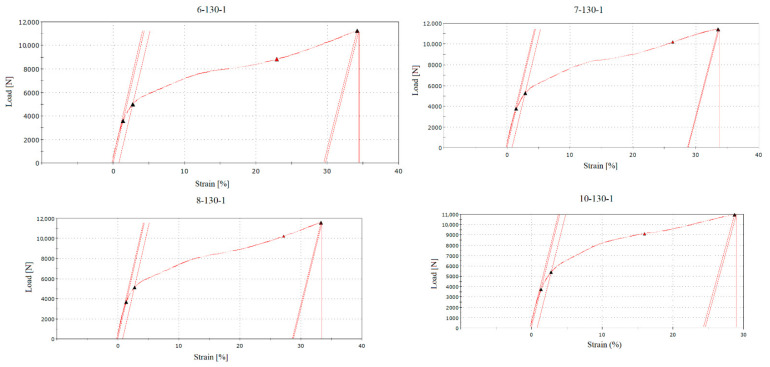
Comparison of samples “6-130-1”, “7-130-1”, “8-130-1”, and “10-130-1” via a tensile diagram with a marked point (red triangle) of RFID tag damage and its deactivation.

During the tests, the reader indicated ongoing communication and reading of information from the RFID tag antenna by means of sound signals and flashing light. Interruption of these indicators also meant interruption of reading the RFID tag, i.e., its damage. Figure 13 characterises the comparison of samples no. 6, 7, 8, and 10 with the missing data of sample “9-130-1”, the measurement of which was unsuccessful, as in the previous case of sample “30-130-0” mentioned in Section 3.1. According to Figure 13, it is clear that the deactivation of the tag was always performed during the second half of the ongoing test. Tensile test of RFID tags type no. 1 (see Figure 7A) proved their usefulness in case of damage detection applications.

### 3.3. Measurements Using Samples with RFID Tags: Type No. 2

The following measurements evaluate tensile tests using RFID tags type no. 2. The difference compared to the first and second measurements is that the RFID tag type no. 2 has a larger dimension (see Section 2.4) and is much thicker than the type 1. Table 3 characterises the average values of measurements while capturing the maximum values of the selected parameters.

**Figure 14:**
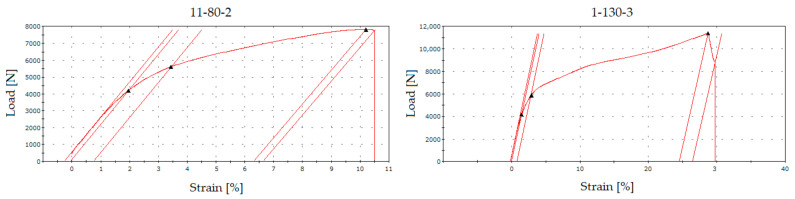
Comparison of tensile tests samples “1-130-2” (**right**) and carriages “11-80-2” (**left**).

The Table 3 shows that none of the measured samples was invalidated by the failed measurement during the tests, since all tests were performed successfully. Within the graphical course of measurements, the results were slightly different, and the samples (11–15) showed a parabolic course of loading against strain. Figure 14 shows the graphical course of the tensile test of sample “1-130-2” (Figure 14 right) and the carriage “11-80-2” (Figure 14 left).

In terms of recording the interruption of the RFID tag signal by the recording antenna, the samples showed no change. The RFID tag was not damaged in any of the cases, as the core and the base of the tag remained in one piece. This fact applies to fabric type “80”. In the case of the “130” fabric, interruptions in the transmission of the RFID tag were captured. However, these phenomena were not significant, as tag antenna damage or transmission interruptions were achieved just before the total destruction of the sample.

**Figure 15:**
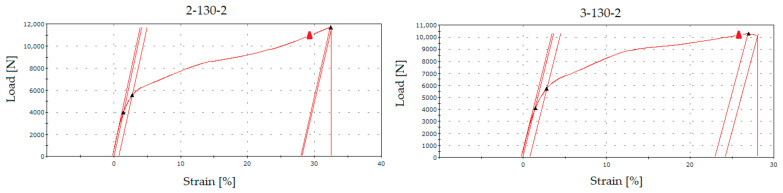
Tensile tests with samples “2-130-2” (**Left**) and “3-130-2” (**Right**) with indication of the point of interruption of transmission (red triangle) of the RFID tag.

This case is unsuitable and unusable in practice. Visualisation of the sample tests with RFID tag type no. 2 and fabric type “130” is done in Figure 15, which combines samples 2 and 3. In the case of sample “1-130-2”, the RFID tag broke, but despite a temporary interruption, it started transmitting again. In the case of sample “5-130-2”, the RFID tag was not broken. The numerical transmission interruption for sample “2-130-2” occurred 208 s after the start of the test, which corresponded to a load value of 10 6398 N and a strain of 34.8 mm. The percentage of strain was then calculated into a graphic interpretation. In the case of sample 3-130-2, the RFID tag was interrupted within 203 s of the start of the test. This time recorded a load value of 10 238 N and an elongation value of 30.2 mm.

From the results of the tests, it can be argued that RFID tags type no. 2 (see Figure 7B) are not suitable for the application of internal crack detection, as confirmed by tensile tests.

### 3.4. Measurements Using Samples with RFID Tags: Type No. 3

Table 4, like the previous case of Table 3, shows that none of the measured samples were invalidated by the failed measurement during the tests. In the case of using the “80” type fabric, no mechanical violation of the RFID tag or interruption of the transmission were recorded. In this respect, the combination of the tag (see Figure 7C) and fabric in the configuration used is unsuitable for further use.

When using a “130” type fabric, similar results were recorded as with the RFID type no. 2 tag (see Figure 7B). Sample “21-130-3” showed an interruption of transmission of two times during the experiment, but even after the rupture of the sample, the tag was still able to transmit. Such results were also recorded for the remaining samples 22–25. In this respect, the RFID tags type no. 3 (see Figure 7C) are also inappropriate for damage detection applications.

### 3.5. Comparison of the Average Values for Individual Measurements

The following section compares the average values of recorder parameters between the different groups of RFID tags and the fibreglass fabrics used. It can be seen from Table 5 that when using the “130” fabric type, the ability to carry a larger load was considerably greater.

The graphic design in the following figure interprets the numerical values from Table 5. According to these results, it becomes evident that the used fabric type “130” shows a considerable degree of elongation, which is not a positive parameter from the measurement results.

**Figure 16:**
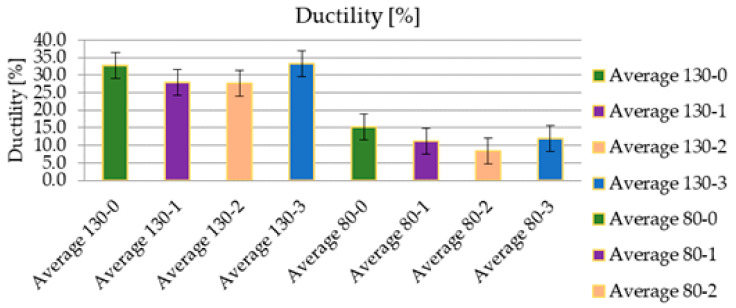
Comparison of average ductility [%] values between the type of RFID tag used and the type of fabric used.

**Figure 17:**
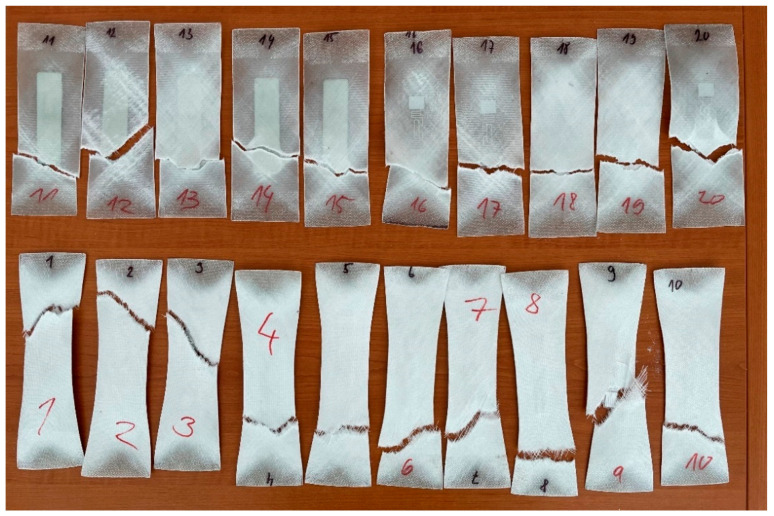
Course of damage for individual tested samples.

The graphical illustration in Figure 16 shows the influence of the implementation of the RFID tag into the structure on its mechanical properties. While the integrity of the “130” fabric was not significantly affected, the “80” fabric was strained at most in the case when the RFID tag was not integrated into the tested sample. The crack initiation has been monitored and analysed during the whole duration of tensile testing, meaning from the moment of applied load, until the complete rupture of the tested samples. Figure 17 collects the most important carriages no. 1–20 and their protruding shape after the test, together with the position of the crack.

In some cases, the sample ruptured at the location of the RFID tag. These were mainly the tag types 2 and 3, but in the case of the type 1 tag, the crack was out of the place. This fact suggests that a given type of RFID tag detected an internal crack that would be more effectively detectable by RFID reading than by NDT diagnostics.

Figure 17 shows samples no. 16-20 in the top row, on the right side, equipped with the RFID tag type no. 1-AZ9662 H3 (see Figure 6A), showed the most significant amount of strain, proving that this type of the RFID tag is the most useful for damage detecting applications among our three tested types. The future research could focus on comparing the different fibre orientations or the use of smaller or larger number of layers inside the laminate structure with this type of passive RFID tag.

**Figure 18:**
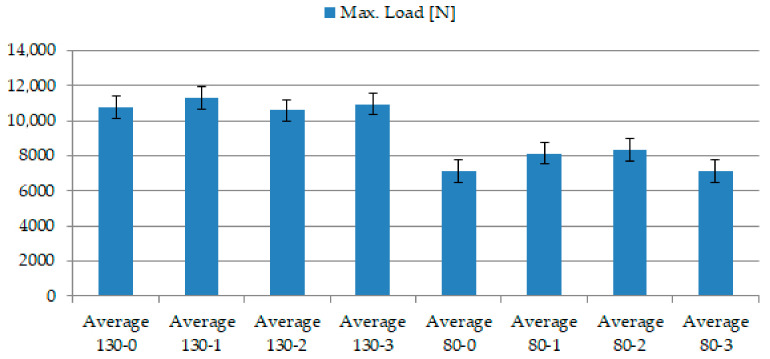
Comparison of average Maximum load [N] values between the type of RFID tag used and the type of fabric used.

Figure 18 compares the average maximum load in [N] values between the type of RFID tag and the fabric type used. In particular, it is a visualisation of the load transfer and the influence of the RFID tag in the samples’ structure, similar to the comparison of ductility in the fabric type and RFID tag used. While in the case of the “130” fabric type, there were roughly similar waveforms, the “80” fabric type showed a larger transferred load with an integrated RFID tag. As this was not the subject of research, it would be appropriate to examine this behaviour in further research. In this case, there is a presumption that an integrated RFID tag could positively change the mechanical properties of the fibreglass patterns used and increase their strength. From this point of view, the integration of the RFID tag into a place with an increased internal stress would not necessarily present an increased risk of cracking.

For this reason, it can be claimed that the application of RFID tags to detect internal cracks is more suitable for fabrics of lower weight and twill type. However, tests have shown that the RFID tag breaks during loading due to internal cracks in the fibreglass structure. Therefore, it can be argued that the implementation of RFID tags in an aircraft structure makes sense and is beneficial in a way that it detects the required parameters.

## 4. Discussion

In the aviation world, there is currently a growing trend of using composite materials in the systems and construction of aircraft from general aviation through airliners to aerospace. With increasing requirements for safety and especially reliability, the current issue is the solution of diagnostics and prediction of fault conditions, or failure-free operation, which would overall affect safety.

The main limitation of our proposed methodology represents the fact, that RFID tags could be implemented only in non-conductive materials, due to creation of adverse closed circuit. Ideal application option represents the fibreglass composites and non-conductive materials used in, e.g., general aviation aircraft (gliders, ultralights, aircraft up to 5400 Kg [42].

The results also showed that the ability to detect structural damage in its early stage is not possible, since interruptions which represented the damage states detected with our proposed method occurred for very high strains—more than 20%.

Since the present article covered only one option of ply orientation set to 45 degrees, it would be worth researching the use of different ply orientations of fibreglass laminates (0°–90°), since, as was mentioned in the introduction chapter, this orientation is related with the stress distribution in composite material. During the experiment, it was found that epoxy resin did not provide sufficient adhesion of the tag to the individual laminated layers, resulting in its undesired shifts, which represents a need for methods which could affect said adhesion [43].

In addition to the use of this technology in integration into fibreglass structures, RFID tags could also be used in the process of “rapid prototyping” or “reverse engineering” using 3D printing. Since 3D printing does not provide a fully homogeneous structure and the use of NDT is also limited to a similar extent as with composites, it is possible to use the detection of internal cracks in this case as well. These are mainly components that have undergone the process of optimising the topology, or as mentioned [44], the lightening of the internal structure to maintain strength. In connection with aviation, the RFID tag can also be used to monitor the technical condition and current maintenance interventions, as well as repairs performed by writing information to the tag itself. Therefore, such use would lead to the reduction of the created paper documentation from the parts, their falsification, or the prevention of the loss of the individual parts’ complete service history.

Lastly, the authors created a comparison of the currently most-used NDT methods for composite materials, namely RT methods, ultrasound, and Eddy Current methods with the presented RFID-based damage detection methodology. The comparison of individual methods is possible through several parameters. Table 6 compares the significant parameters of individual methods, such as whether there is contact with the tested material during the inspection or not, time needed to perform the method, the accessibility of the method and their complexity due to specialisation requirements, application time, and approximate price range, to highlight the general pros and cons of the presented methodology.

## 5. Conclusions

The research provided a conclusion for the most appropriate type of RFID tag used. Among three tested types of passive RFID tags, only one type has proved to have sufficient properties for structural monitoring applications. The type which proved to be the most useful for antenna deformation-based damage detection was RFID tag type no. 1-AZ9662 H3 (see Figure 6A).

Basic research opened the door to further applied research in the scope of the effective implementation of tags in composite structures to reduce the relative elongation at the point of tag deformation to detect very minor damage. The solution provides an innovative approach to monitoring internal cracks without the use of costly methods, such as RT, Ultrasound, or Eddy Current, which require trained staff, a timeline for access to measuring points, and a special workplace. Placement of the RFID tags in critical places, such as places with high voltage concentration, places exposed to aggressive environments (salts, oils, liquids, and temperature extremes), or places exposed to surface stress, abrasion, or cyclic stress and vibration.

The aim is to point out the possible usage of RFID tags as one of the forms of non-destructive testing of aircraft composite structures. The results of our presented methodology showed its effectiveness in case of identifying damage (cracks) in the later stages of its development. Based on the findings, it is possible to propose the inclusion of RFID-based checks among common individual type of maintenance inspections. In the case of the inclusion of low cost and reliable RFID checks into inspection processes of the line maintenance, this could to some extent cover the tasks which would be otherwise part of the scheduled heavy maintenance (the D check). This could increase the time required to perform the line maintenance tasks, but on the other hand, it could detect damages or failures, which could be detectable only during heavy maintenance, leading to direct impact on safety enhancement.

In addition to damage detection ability, the RFID tags could work as a storage device suitable for recording the damage history and its development over time.

The secondary results and conclusions of the research were the fact that the integration of the samples into the structure of the fibreglass samples did not affect the overall strength and the reduction of the composite samples’ mechanical properties. Thus, the analysis of mechanical tests determined the suitability of using RFID tags to determine internal cracks for a chosen tag type, and for the type of fibreglass fabric and the direction of the loading force against the orientation of the fibres. The research provided new scientific questions for the creation of further experiments that should address the system of increasing the adhesion between the surface of the RFID tag and the internal structure of the composite materials (individual layers), as well as the orientation of the fibres towards the load.

The manuscript provided an insight into the possibilities of using RFID technology in the detection of internal cracks in composite materials, and based on the results of research, it can be argued that the technology could be used as a form of NDT diagnostics in the maintenance process. The use of RFID for a particular application, therefore, provides opportunities to increase the safety and failure-free operation of an aircraft and provides new opportunities to optimise maintenance programs.

## Figures and Tables

**Table 1:** Average values of mechanical parameters of the samples without an integrated RFID tag.

	Sample No.	Max. Load [N]	Ultimate Tensile Strength Rm [MPa]	Proof Stress Rp 0.2 [MPa]	Ductility [%]
	26-130-0	11,364	122	36	37.3
	27-130-0	12,162	140	39	34.1
	28-130-0	9477	111	38	28.9
	29-130-0	10,046	118	39	30.6
	30-130-0	-	-	-	-
Average		10,762	123	38	32.7
	36-80-0	7442	105	46	18.1
	37-80-0	7100	100	44	16.9
	38-80-0	6358	90	44	10.7
	39-80-0	7199	102	46	15.0
	40-80-0	7581	106	38	16.0
Average		7136	101	44	15.3

**Table 2:** Average values of the mechanical parameters of samples with integrated RFID tag type no. 1.

	Sample No.	Max. Load [N]	Ultimate Tensile Strength Rm [MPa]	Proof Stress Rp 0.2 [MPa]	Ductility [%]
	6-130-1	11,226	135	43	29.8
	7-130-1	11,447	136	45	28.8
	8-130-1	11,595	136	43	28.7
	9-130-1	-	-	-	-
	10-130-1	10,936	134	46	24.5
Average		11,301	135	44	28.0
	16-80-1	109	53	53	9.8
	17-80-1	116	56	56	8.7
	18-80-1	121	56	56	14.5
	19-80-1	123	57	57	14.0
	20-80-1	112	54	54	9.2
Average		116	55	55	11.2

**Table 3:** Average values of mechanical parameters of samples with integrated RFID tag type no. 2.

	Sample No.	Max. Load [N]	Ultimate Tensile Strength Rm [MPa]	Proof Stress Rp 0.2 [MPa]	Ductility [%]
	1-130-2	11,378	137	50	26.4
	2-130-2	11,704	137	47	28.1
	3-130-2	10,310	112	44	24.2
	4-130-2	9545	114	43	27.4
	5-130-2	9918	121	42	32.8
Average		10,571	124	45	27.8
	11-80-2	7819	109	58	6.6
	12-80-2	7627	107	54	8.7
	13-80-2	8651	119	57	11.7
	14-80-2	8387	112	54	7.5
	15-80-2	9219	129	63	7.4
Average		8341	115	57	8.4

**Table 4:** Average values of mechanical parameters of samples with integrated RFID tag type no. 3.

	Sample No.	Max. Load [N]	Ultimate Tensile Strength Rm [MPa]	Proof Stress Rp 0.2 [MPa]	Ductility [%]
	21-130-3	11,015	124	41	31.2
	22-130-3	11,307	128	39	32.4
	23-130-3	10,703	119	41	28.8
	24-130-3	11,303	127	38	33.7
	25-130-3	10,398	113	37	40.1
Average		10,945	122	39	33.2
	31-80-3	6854	92	45	11.8
	32-80-3	8047	107	50	15.6
	33-80-3	6894	95	47	11.6
	34-80-3	6877	97	48	12.8
	35-80-3	7008	98	52	8.1
Average		7136	98	48	12.0

**Table 5:** Summary of the average values of mechanical parameters of samples type no. 0 to no. 3.

Samples Group	Max. Load [N]	Ultimate Tensile Strength Rm [MPa]	Proof Stress Rp 0.2 [MPa]	Ductility [%]
Average 130-0	10,762	123	38	32.7
Average 130-1	11,301	135	44	28.0
Average 130-2	10,571	124	45	27.8
Average 130-3	10,945	122	39	33.2
Average 80-0	7136	101	44	15.3
Average 80-1	8141	116	55	11.2
Average 80-2	8341	115	57	8.4
Average 80-3	7136	98	48	12.0

**Table 6:** Comparison of commonly used NDT methods for composite materials with the proposed RFID-based method; specifications based on [4,13,16,45,46,47,48,49].

Method or Approach Used	Passive RFID Tags	Visual Inspection	Ultrasonic Method	Radiographic Testing (RT)	Capillary Liquid Penetrant	Eddy Current Testing
**Key Abilities**	Wireless internal damage detection of covered areas	Visual surface and internal structure inspection based on observer’s subjective assessment	PC	Uses electromagnetic shortwave radiation to illuminate the tested materials	Surface visual inspection based on subjective assessment by the observer	Uses electromagnetic induction to inspect tested materials
**Time to Perform**	In under	Up to 10	Up to 1 h	Up to 1 h	Up to 1 h	Up to 1 h
**Usage Efficiency**	Low/Medium	Low/Medium	High	High	Medium	High
**Interrogation Distance**	5–500 mm	-	5–10 mm	5–400 mm	-	-
**Investigation Area Size**	Depends on covered area size and number of tags used	Not limited	Limited by the accessibility of NDT technology	Limited by the accessibility of NDT technology	Not limited	Limited by the accessibility of NDT technology
**Presence of Contact**	Non-Contact	Non-Contact	Non-Contact	Non-Contact	Contact	Contact
**Price Range**	Affordable method	Affordable method	Expensive method	Expensive method	Affordable method	Expensive method
**Performed as a task of**	Line Maintenance	Heavy Maint. (D check)	Heavy Maint. (D check)	Heavy Maint. (D check)	Heavy Maint. (D check)	Heavy Maint. (D check)
